# Phenomics Research on Coronary Heart Disease Based on Human Phenotype Ontology

**DOI:** 10.1155/2014/240284

**Published:** 2014-12-15

**Authors:** Qi Shi, Kuo Gao, Huihui Zhao, Juan Wang, Xing Zhai, Peng Lu, Jianxin Chen, Wei Wang

**Affiliations:** ^1^The Key Institute of State Administration of Traditional Chinese Medicine (Pneumonopathy Chronic Cough and Dyspnea), China-Japan Friendship Hospital, Beijing 100029, China; ^2^Beijing University of Chinese Medicine, Bei San Huan East Road, Beijing 100029, China; ^3^Institute of Automation, Chinese Academy of Sciences, Zhongguancun East Road, Beijing 100190, China

## Abstract

The characteristics of holistic, dynamics, complexity, and spatial and temporal features enable “Omics” and theories of TCM to interlink with each other. HPO, namely, “characterization,” can be understood as a sorting and generalization of the manifestations shown by people with diseases on the basis of the phenomics. Syndrome is the overall “manifestation” of human body pathological and physiological changes expressed by four diagnostic methods' information. The four diagnostic methods' data could be the most objective and direct manifestations of human body under morbid conditions. In this aspect, it is consistent with the connation of “characterization.” Meanwhile, the four diagnostic methods' data also equip us with features of characterization in HPO. In our study, we compared 107 pieces of four diagnostic methods' information with the “characterization database” to further analyze data of four diagnostic methods' characterization in accordance with the common characteristics of four diagnostic methods' information and characterization and integrated 107 pieces of four diagnostic methods' data to relevant items in HPO and finished the expansion of characterization information in HPO.

## 1. Background

Since the early 1990s, “omics” began to emerge in the field of life sciences as the most compelling concept, including genomics, proteomics, transcriptomics, and metabolomics. The characteristics of holistic, dynamics, complexity, and spatial and temporal features enable “omics” and theories of TCM to interlink with each other. The holistic and systematic ideology of TCM is reflected at a macro level, while the omics technology is the reintegration based on comprehensive analysis and restoration. Omics technology helps to quantify, objectify, and standardize TCM theory and further provides a new platform to evidence-based researches on TCM theories.

With the rise and development of different types of “omics,” in 1996, Steven A. Garan, Director of Aging Research Center, first put forward the concept of “phenomics” in a lecture delivered at the University of Waterloo. Phenome refers to all the phenotypes of a cell or an organism, whose life activities are reflected through phenotypes. Phenomics is a subject studying all phenotypes of an organism or a cell in various conditions at the genomic level [1, 2]. Sensitive Phenomics research tools can reveal a number of intermediate phenotypes which are previously unknown, and they are expected to be applied to the treatment of diseases [3].

The HPO was initially developed using information from OMIM, which is an important data resource in the field of human genetics and beyond. HPO is being developed in an effort to provide a standardized, controlled vocabulary that allows phenotypic information to be described in an unambiguous fashion in medical publications and databases. The HPO is currently using information from OMIM and medical literature. It contains approximately 10,000 terms and over 50,000 annotations to hereditary diseases are available for download. Terms in HPO describe a phenotypic abnormality, such as atrial septal defect. The HPO aims to provide a standardized vocabulary of phenotypic abnormalities encountered in human disease [4].

Phenotype is not only the combination of all the phenomena and features which are manifested; but it also can reflect the essence or nature of things by some means or information. Characterization is widely used in various disciplines. Wright and his team came up with Human Face Recognition Model based on sparse characterizations [5]. A research [6] led by Liu et al. characterized the similarities and differences of metabolic networks of rats between the control group and model group by applying metabonomics methods from the micro perspective in vivo; meanwhile it achieved a phenotype of the traditional Chinese materia medica of cold or warm nature (medicinal properties of Chinese medicine refer to its nature of therapeutic effects, which could be summarized as four properties and five tastes, channel tropism, lift ups and downs, and toxicity; and “cold” and “hot” are an aspect of Chinese medicine medicinal properties). Academician Wang Yongyan believes that, for pattern of blood stasis (blood stasis syndrome is one of the types of TCM syndrome), the manifestations of tongue, lips, hands, helix, ear tips, eyes, hairs, and animal behaviors can all be regarded as the phenotypes of the four diagnostic methods. Accordingly, he put forward the concept of “Characterization Model” [7]. As the researches of characterization model move along, the concept of characterization gradually expanded from the original animal signs and autonomous behaviors to characterization of biological information. It finally achieved the combination of disease model and model of TCM patterns and syndromes and enabled an effective evaluation on these models [8, 9].

TCM follows the basic principle of pattern identification and syndrome differentiation in its clinical practice and believes that accurate differentiation of syndromes is a prerequisite for effective treatment. The called “treatment based on syndrome differentiation” is the basic principle to recognize and treat the disease, and a kind of special researching and treating method for disease in traditional Chinese medicine, including two processes of syndrome differentiation and treatment. TCM syndrome is a kind of pathological generalization of the disease at a certain stage during its development. Because TCM syndrome includes the ill issues, causes, property and the relationship between healthy energy and evil struggle, reflecting the essence of pathological changes of disease development at certain stages, so it could reveal the nature of the disease more comprehensive than simple symptoms. Four diagnostic methods' information included inspection, listening and smelling, interrogation, and pulse feeling and palpation. Syndrome is the overall “manifestation” of human body pathological and physiological changes which can be expressed by four diagnostic methods' information; for example, tongue picture is not only the most important indicator of symptoms and disease patterns of human body, but also the external manifestation of overall body conditions [10]. Our preliminary studies have suggested that using data mining method, like complex network, as a medium study method can effectively achieve the integration of information collected by four diagnostic methods' information of patients with CHD and finally enable the recognition of disease patterns [11, 12]. The four diagnostic data can not only reflect the TCM holistic theory of “there must be external manifestations of internal diseases,” but also be the most objective and direct manifestations of human body under morbid conditions. In this aspect, it is consistent with the connotation of “phenotypes.” Meanwhile, the four diagnostic methods' data also have the features of characterization in HPO, for example, the four diagnostic methods' information, with a large number of complex data content, sharing the general characteristics of structural features and close connection on the basis of the specific nature of its own evolution mechanism. We hope that our work can be a new and deeper interpretation of impact of the four diagnostic methods' information on identification of TCM disease pattern and syndrome from the perspective of Phenomics.

## 2. Materials and Methods

### 2.1. Data Acquisition

#### 2.1.1. Source of Cases

We selected 385 CHD cases with UAP admitted by Dongzhimen Hospital, which is the affiliated hospital of Beijing University of Chinese Medicine, China-Japan Friendship Hospital, Beijing Anzhen Hospital, Zhengzhou Hospital of Traditional Chinese Medicine, Wuhan Hospital of Traditional Chinese Medicine, and Hubei Hospital of Traditional Chinese Medicine, from May 2009 to March 2011.

Other 1665 cases in the follow-up study were selected from patients with CHD and UAP hospitalizing from September 2008 to March 2011, in Dongzhimen Affiliated Hospital of Beijing University of Chinese Medicine, East Hospital, Ministry of Health directly supervised China-Japan Friendship Hospital, Wuhan Hospital of Chinese Medicine, Hospital of Zhengzhou City, Hubei Hospital of Traditional Chinese Medicine, Affiliated Hospital of Liaoning University of Traditional Chinese Medicine, Affiliated Hospital of Traditional Chinese Medicine of Xinjiang Medical University, Hangzhou Hospital of Traditional Chinese Medicine, Chengdu Hospital of Traditional Chinese Medicine and Western Medicine, Affiliated Hospital of Inner Mongolia Medical College, Yichang Hospital of Traditional Chinese Medicine, Henan Hospital of Traditional Chinese Medicine, Wuxi Hospital of Traditional Chinese Medicine, Zibo Hospital of Traditional Chinese Medicine, and Cardiology Department of Changzhou Hospital of Traditional Chinese Medicine.

#### 2.1.2. Disease and Diagnostic Criteria

The diagnostic criteria of angina pectoris was referred to the Treatment Guidelines Chronic Stable Angina (1999) jointly identified by American College of Cardiology (ACC)/American Heart Association (AHA)/American College of Physicians and the American Society of Internal Medicine ACP-ASIM [13], and the Diagnosis and Treatment Recommendations of Unstable Angina made by Chinese Medical Society of Cardiology in 2000 [14].

The TCM clinical diagnosis criteria of angina pectoris was referred to chapter of syndrome and pattern in* Terminology of TCM Clinical Diagnosis Criteria *made by the Chinese National Technical Supervision Administration in 1997 [15]; guidelines for clinical research on TCM treatment of thoracic obstruction (CHD angina pectoris) in* Guidelines of Chinese Medicine Clinical Research on TCM New Drugs* published in 2002 can serve as reference of TCM pattern identification [16].

#### 2.1.3. Inclusion Criteria, Exclusion Criteria, and Eliminated Criteria

*Inclusion Criteria*. (1) Hospitalized patients between the ages of 20 and 90, male or female; (2) patients who met the diagnostic criteria of UAP; (3) patients who voluntarily signed the informed consent.

*Exclusion Criteria*. (1) Patients with acute myocardial infarction, myocarditis, pericardial disease, cardiac neurosis, intercostal neuralgia, menopausal syndrome, and severe chest pain caused by cervical spondylosis; (2) patients with angina caused by other diseases, such as rheumatic fever, syphilis, congenital coronary deformity, hypertrophic cardiomyopathy, aortic stenosis, or regurgitation; (3) patients with combined stroke, pulmonary infection, nephritis, renal failure, urinary tract infections, rheumatism, heart failure, severe arrhythmias, cancer, and severe primary diseases of liver, kidney, and hematopoietic system; patients with heart disease history, whose systolic blood pressure ≥ 180 mmHg and/or diastolic blood pressure ≥ 110 mmHg before or after treatment control; (4) patients who were pregnant or in breast-feeding period; (5) allergic patients; (6) mentally ill patients. 

*Eliminated*. (1) Patients violating inclusion criteria or meeting with exclusion criteria; (2) patients whose data could not be collected or analyzed because of inadequate clinical data after being included.

#### 2.1.4. Clinical Data Collection

General information, history of present illness, past history, personal illness, family disease history, and other related symptoms of patients meeting the inclusion criteria with UAP and CHD were gathered within 24 hours of admission to make a detailed record of four diagnostic methods' information data and complete the four clinical diagnostic information collection forms. The collection of four diagnostic methods' extended phenotypes served as indicators of the above five TCM patterns information and patients' disease history must be done by professionals, who must meet with at least one of the following specific requirements: physicians with professional qualification; attending physician or above; and personnel with two years of relevant clinical experience or more; postgraduate students of relevant majors in their first year or above can only participate under the supervision of attending physicians or above.

### 2.2. Research Methods

#### 2.2.1. Syndrome Differentiation

Within 24 hours of admission, three deputy chief physicians with 5 years or more of clinical experience in relevant field would give judgments on TCM syndromes/syndrome factors (the complex TCM syndrome is decomposed into more limited elements, with more relatively clear content, which is called the TCM “syndrome elements”) and syndrome identification based on previous diagnosis of patients. Identification criteria of syndrome/syndrome factors are as follows: (1) identification can be made when the three physicians hold the same opinion; (2) when one physician disagrees with the other two, the opinion of the latter two would be adopted; (3) when the 3 physicians hold different opinions against each other, the deputy director or above can recollect information obtained by the four diagnostic methods' information and identify syndrome, syndrome factors, and disease pattern all over again until it meets with the first and second situation.

#### 2.2.2. Observation Methods of Tongue Picture and Sublingual Collaterals

Tongue pictures of 212 patients were taken by a digital camera (RICOH, R10, 10 million pixels) after 10:00 a.m. before lunch. Before shooting, patients would rise 2 to 3 times with warm water and have a 15-minute rest. When pictures were taken under natural light, patients would keep in a sitting position against the light and roll their tongue to the largest extent to expose their sublingual collaterals (there are two longitudinal veins on each side of the frenum linguae under the tongue, which is called “sublingual collaterals” in TCM).

The camera would be backlit during shooting, turning off the flash while using colorimetric cards. The color cards were close to the patients' tongue and maintained the same level with tongue. The camera and color card were kept in a vertical position. The focal point lied in the junction of the tongue and the colorimetric cards. Photos of tongue picture included visible color cards and full view of sublingual collaterals. The time limit of patients sticking out their tongue should not exceed 15 seconds every time. Shooting of tongue pictures would resume after a 5-minute break. There must be no less than three clear pictures of sublingual collaterals for each patient.

Observation measures of sublingual collaterals can refer to the formulation that sublingual collaterals can be described from the following four aspects: length, thickness, tortuosity, and color in* Diagnostics of Chinese Medicine* [17], textbook of colleges and universities of traditional Chinese medicine. Accordingly, in our study, sublingual collaterals were observed on the following indicators: collaterals' length was divided into three categories: long (longer than 3/5 of the connection of tongue and sublingual caruncle), medium long (length roughly equals 3/5 of the tongue and sublingual caruncle connection), and short (shorter than 3/5 of the tongue and sublingual caruncle connection); thickness of collaterals was divided into three degrees: thick (root diameter exceeds 2.7 mm), medium thick (root diameter ranges from 1.5 mm to 2.7 mm), and fine (root diameter less than 1.5 mm); tortuosity of sublingual collaterals was divided into three degrees: mild (no obvious or slight bending of trunk collaterals), moderate (obvious trunk bending), and severe (trunk severely bent); collaterals color was divided into purple, dark purple, and black purple.

#### 2.2.3. Sources of Four Diagnostic Methods' Data

107 pieces of four diagnostic methods' information gathered from clinical epidemiological surveys on the UAP patients mentioned above were used as data sources, including chest pain, chest distress, short breath, palpitation, cough, aversion to cold and cold extremities, fatigue and weakness, spontaneous perspiration, night sweat, burning sensation of five centers, eyestrain, dry mouth, dizziness, amnesia, fainting feeling, tinnitus, dry and hot face, insomnia, irascibility, hypochondrium distending pain, sighing, depression, anorexia, abdominal distension, epigastric fullness, belching, nausea and vomiting, loose stool, constipation, sore lumbus and knees, frequency of micturition at night, limb numbness, heel pain, hemiplegia, subcutaneous ecchymosis, pachylosis, obesity, white phlegm, yellow phlegm, bloody phlegm, frothy sputum, pharyngeal foreign body sensation, thirst with no desire for drinks, tastelessness, bitter taste in the mouth, sweet taste in the mouth, salty taste in the mouth, viscous and greasy taste in the mouth, diarrhea, defecation asthenia, yellow urine and oliguria, clear urine in large amounts, residual urine, cold abdomen and waist, heavy limbs, pallor, bright pale complexion, darkish complexion, sallow complexion, red complexion, conjunctiva congestion, dark color around eyes, edema of the eyelids, dark red lip and gingival, pale lips and finger nails, dark color in palatal mucosa, lower abdominal tenderness, lower extremity edema, faint low voice, emaciation, pale tongue, red tongue, scarlet tongue, dark red tongue, dark purple tongue, swollen tongue body, thin tongue body, tooth-marked tongue, thick tongue coating, greasy tongue coating, curry tongue coating, scanty tongue coating, exfoliative tongue coating, no tongue coating, thick and greasy tongue coating, smooth tongue coating, yellow tongue coating, black tongue coating, glossal petechia, lavender subglossal collateral vessels, blue purple subglossal collateral vessels, black purple subglossal collateral vessels, mauve subglossal collateral vessels, faint red subglossal collateral vessels, subglossal collateral vessels engorgement, thin subglossal collateral vessels, deep pulse, rapid pulse, moderate pulse, knotted and intermittent pulse, slow pulse, thread pulse, wiry pulse, tense pulse, uneven pulse, smooth pulse, and weak pulse.

#### 2.2.4. Data Processing and Methods

*Validation Methods and Establishment of Decision Tree Model Based on Four Diagnostic Methods' Information*. 30 pieces of four diagnostic methods' information with statistical significance tested and analyzed by SPSS17.0 t were entered into the screening process of decision tree model as independent variables, while “with or without blood stasis pattern” was entered as a dependent variable. The entering order of 30 pieces four diagnostic methods' data was in accordance with the degree of statistical significance showed by* t*-test. SPSS17.0 CHAID, QUEST tree, Weka3.5.5 J48, and decision tree model were used to explore diagnostic rules. Sample volume limitation was taken into consideration in the construction process of CHAID and QUEST model. In order to ensure and promote the development of decision tree model, set 50 as the parent node, 25 as child node. When building the C4.5 and AD decision tree model, system defaults including J48-C 0.25-M 2 and ADTree-B10-E-3 were applied to all.

10-fold cross-validation method was applied to verify the decision tree model of recognition mode. TP represented the identification of the correct number of samples with blood stasis pattern; TF represented the correct recognition of number of samples without blood stasis pattern; FN meant the false identification of the number of samples with blood stasis pattern; FP meant misidentification of samples without blood stasis pattern [18].

*Clustering Analysis Methods and Comparison between the Four Diagnostic Methods' Information and Phenotypes*. 107 pieces of four diagnostic methods' information were compared with OMIM information of human body superficial characteristics from the OMIM Database (OMIM—Online Mendelian Inheritance in Man, http://www.omim.org/). MetaboAnalyst 2.0 software (retrieved from http://www.metaboanalyst.ca/MetaboAnalyst/) was used as a tool to conduct cluster analysis of the four diagnostic methods' which were identical.

*Complex Network Analysis Methods of 107 Four Diagnostic Methods' Information Based on 23 Characteristics*. Processing and entering of data: Mutual information method was applied to set up the relationship between the information nodes of 107 pieces of four diagnostic methods' information of 2,050 cases of UAP, setting the weight of 1. We organized the above collection of data to be an adjacency matrix and then converted it into the format required by Pajek software and finally formed complex network map of four diagnostic methods' information in patients with CHD and UAP. The connectivity analysis and impact analysis were made by using Pajek 2.0 analysis software. The impact value and connectivity were, respectively, calculated. Commands of Layout-Energy-Kamada-Kawai-Separate Components and Operation-Shrink Network-Partitions were applied in drawing different categories/weights node map, with assistance of the manual mediation of the nodes. Adjustment principles were as follows: removing isolated nodes, adjusting nodes position, instead of deleting edges and remaining nodes. We constantly improved the four diagnostic information methods' map which was outputted by Bitmap format.

*Method for Classifying the 107 Four Diagnostic Methods' Information Items into HPO*. Firstly, we read the related items in the HPO, finding that these items were classified according to different systems, disease location, and clinical symptoms. Then the 107 items had been classified according to HPO, which were showed in the attached Excel file (in Supplementary Material available online at http://dx.doi.org/10.1155/2014/240284). In the Excel file, “TCM Representation” represents Chinese name of 107 four diagnostic methods' information items; “English Items” represents English name of 107 four diagnostic methods' information items; “Father Node” represents the original item names of HPO, and the detailed classification method has been described; the corresponding figures of “Father Node” had been shown at the last row in the Excel file. For example, there is original item “angina pectoris” in HPO, and the “chest pain,” “chest distress,” and “short breath” are the most common symptoms for “angina pectoris,” so they had been classified into “angina pectoris” (Line 2–Line 4 in the Excel). The 107 four diagnostic methods' information items could be classified into HPO by 6 kinds of methods, which had been described detailedly at the section of “Results—HPO categorization results of 107 pieces of four diagnostic methods' information.”

## 3. Results

### 3.1. Distribution of Sublingual Collaterals for Different Patterns

**Figure 1:**
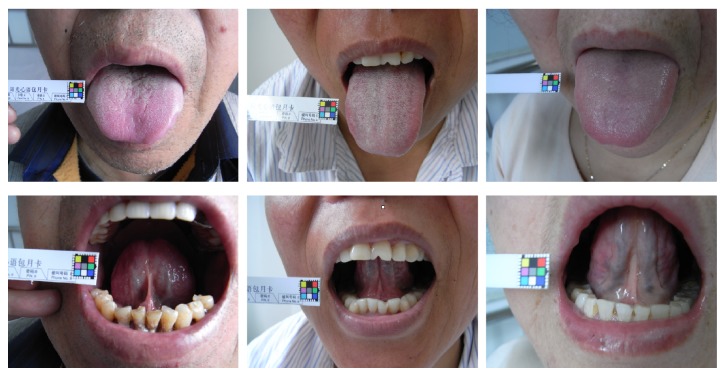
Pictures of tongue surface and sublingual collaterals gathered from patients with UAP.

Short, thin sublingual collaterals with mild and moderate tortuosity, showing a color of mild purple, could be seen in patients with pattern of congealing cold in heart vessel and pattern of phlegm obstruction in heart vessel; long, thick sublingual collaterals with medium or severe tortuosity, manifesting a color of dark purple or black purple, could be seen in patients with pattern of blood stagnation and pattern of blood obstruction in heart vessel; short, medium thin sublingual collaterals with mild tortuosity, exhibiting a color of mild purple, could be seen in patients with deficiency pattern, such as pattern of mutual deficiency of yin and qi, pattern of heart qi deficiency, heart yang deficiency pattern, and heart yin deficiency pattern. The results were shown in Table 1, Figure 1.

### 3.2. Decision Tree Model of Blood Stasis Recognition Based on the Characterization of the Four Diagnostic Methods' Information

**Figure 2:**
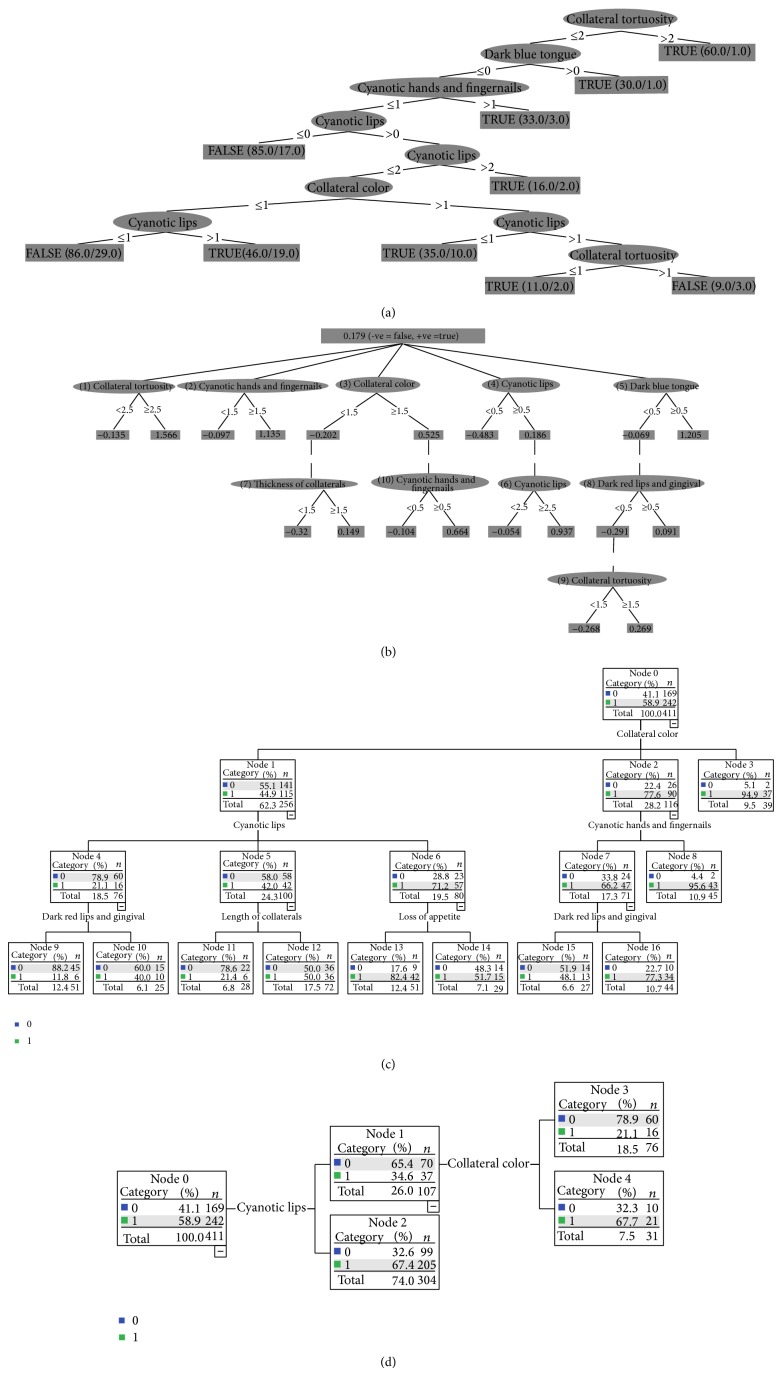
Decision tree model of blood stasis pattern identification for 411 cases of UAP. (a) C4.5 decision tree model; (b) ADTree decision tree model; (c) CHAID decision tree model; (d) QUEST decision tree model.

Variables and corresponding values selected by *t*-test screening are shown in Table 2: 0, 1, 2, 3, respectively, represent no symptoms, with symptoms of mild, moderate, and severe degree; 0, 1 stand for with and without symptoms. Six attributes, including collaterals' color, cyanotic lips, cyanotic hands and fingernails, tortuous collaterals, dark blue tongue, dark red lips, and gingival were filtered out by C4.5 algorithms and altogether formed one decision tree model. There are 10 leaf nodes in the model forming 10 diagnostic rules of blood stasis pattern. TRUE means blood stasis syndrome; “FALSE” stands for non-blood stasis syndrome. Seven attributes are filtered out by the ADTree algorithms after screening, including cyanotic lips, cyanotic hands and fingernails, tortuous collaterals, dark blue tongue, dark red lips, and gingival and thickness of collaterals, altogether forming another decision tree model. There are 21 leaf nodes in the model. TRUE means blood stasis syndrome. Collaterals' tortuosity and cyanosis level of hands, fingernails, and tongue can serve as direct indicators of blood stasis pattern. Six attributes, including collaterals' color, cyanotic lips, collaterals' length, loss of appetite, cyanotic hands and fingernails, dark red lips, and gingival are selected by CHAID algorithm after screening 30 pieces of four diagnostic methods' information. The depth of this model is 3. There are 17 nodes in total, of which 10 end nodes form 10 pairs of identification routes of blood stasis pattern formation. Thus it can be seen in the decision tree models that color of sublingual collaterals is the best variable to identify blood stasis pattern. For cases with black blue sublingual collaterals, this property is the only effective variable of blood stasis pattern identification and its accuracy rate has reached 94.9%. Seven attributes including cyanotic lips, collaterals' color, cyanotic hands and fingernails, tortuous collaterals, dark blue tongue, fatigue, and loss of appetite are selected by SPSS17.0 QUEST algorithm after screening. The depth of this model is 2 and there are a total of five nodes, of which three end nodes form three pairs of identification routes of blood stasis pattern. In this mode, the attribute of cyanotic lips is the best variables to identify blood stasis pattern (see Figure 2). Verified by the 10-fold cross-validation method, the accuracy rates of the above four models, C4.5, ADTree, CHAID, and QUEST model, were 76.7%, 75.7%, 73.7%, and 69.6%, respectively; and the specificity rates were 72.8%, 75.7%, 78.1%, and 35.5%, respectively; and the sensitivity rates were 77.7%, 75.6%, 70.7%, and 93.4%, respectively (see Table 3).

### 3.3. Cluster Analysis of Characterization Information

Compared with the characterization database, 23 of 107 pieces of four diagnostic methods' information were consistent with it, namely, chest pain, heart palpitations, chills, fatigue, aversion to cold and cold limbs, spontaneous perspiration, night sweats, dysphoria in chest, palms and soles, dry eyes and mouth, dizziness, irritability and impatience, lack of tongue moss, depression, loss of appetite, bloating, nausea and vomiting, loose stools, nocturia, numbness, bruising, eyelid edema, lower limb edema, and tinnitus (see Table 4).

**Figure 3:**
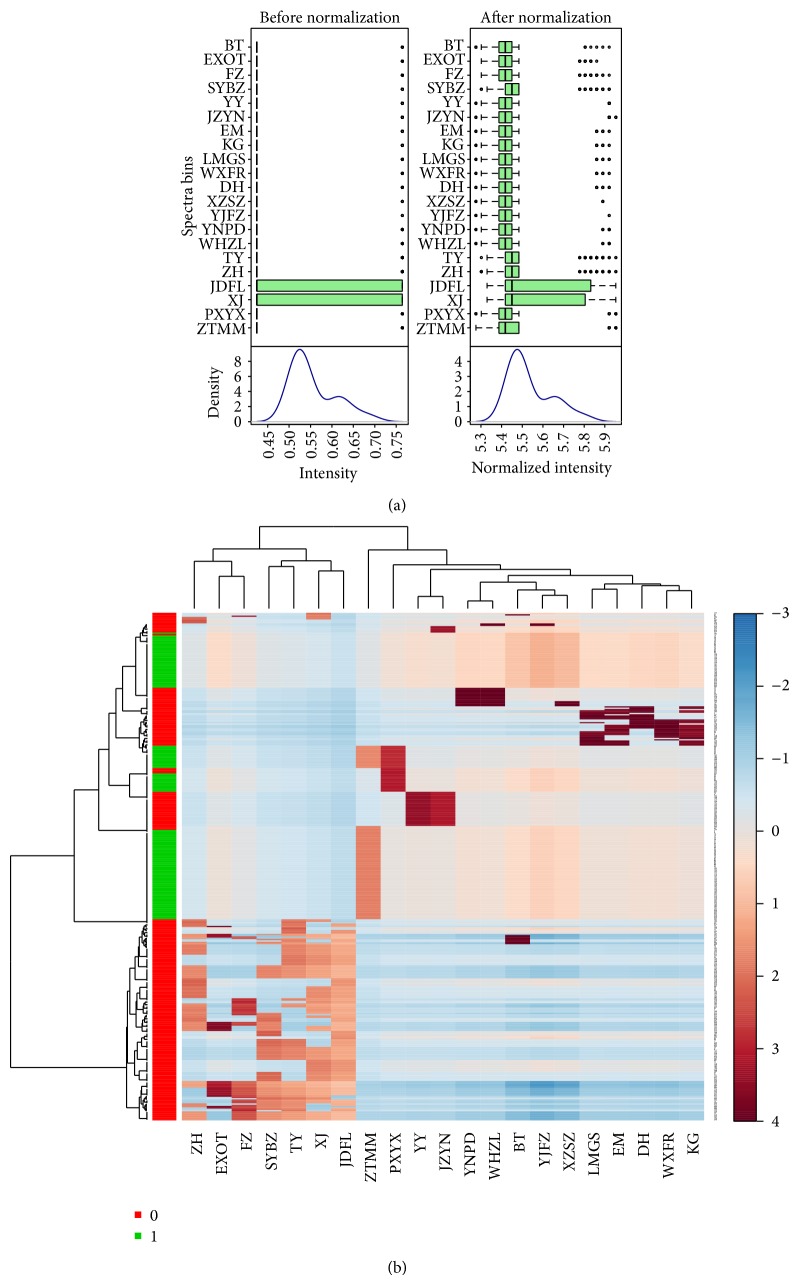
Cluster analyses of 23 characterizations. (a) Data standardization chart; (b) cluster analysis heat map; ZH = spontaneous, EXOT = nausea, vomiting, FZ = bloating, SYBZ = loss of appetite, TY = dizziness, XJ = palpitations, JDFL = lassitude, ZTMM = numbness, PXYX = subcutaneous bleeding, YY = depression, JZYN = irritability, YNPD = nocturia, WHZL = chills and cold limbs, BT = loose stools, YJFZ = eyelid edema, XZSZ = lower extremity edema, LMGS = dry eyes, EM = tinnitus, DH = night sweats, WXFR = dysphoria in chest, palms and soles, and KG = dry mouth.

Hierarchical clustering is commonly used for unsupervised clustering. Agglomerative hierarchical clustering begins with each sample as separate cluster and then proceeds to combine them until all samples belong to one cluster. The result is usually presented as a dendrogram or a heat map. Cluster analysis showed that 21 of the above 23 four diagnostic methods' information had automatically entered the cluster analysis, with the exclusion of “chest pain” and “less moss.” 21 four diagnostic methods' information were aggregated into five parts and the polymerization was good. These five parts prompted five kinds of Chinese medicine syndrome/syndrome elements, respectively, after analysis: spontaneous sweating, nausea, vomiting, bloating, loss of appetite, dizziness, palpitations, and lassitude, all of which aggregated into “qi deficiency” pattern; numbness and subcutaneous stasis blood aggregated into “blood stasis” syndrome pattern; depression, irritability, and impatience aggregated into “qi stagnation” pattern; nocturia, chills, loose stools, eyelid edema, and lower limb edema aggregated into “yang deficiency” pattern; dry eyes, tinnitus, night sweats, dysphoria in chest, palms and soles, and dry mouth aggregated into pattern of “yin deficiency” (see Figure 3).

### 3.4. Complex Network Analysis of 107 Phenotypes

Based on the identification of five TCM patterns, we expanded the sample volume to 2,050 and extended the phenotypes from 21 to 107. We did a complex network analysis on these 107 pieces of information. The results showed that the center of complex network node classification chart was composed of 21 pieces of four diagnostic methods' information, which were consistent with the characterization database, and different colors were assigned to represent five kinds of patterns, namely, pattern of qi deficiency, of blood stasis, of qi stagnation, and of yin and yang deficiency. There were 71 pieces of extended phenotypes served as indicators for the above five TCM patterns, which were arranged in order of priority around the center of network. There were 15 more phenotypes which were not involved into complex network, including emaciation, pale tongue, and rapid pulse, and their information node was 0.

**Figure 4:**
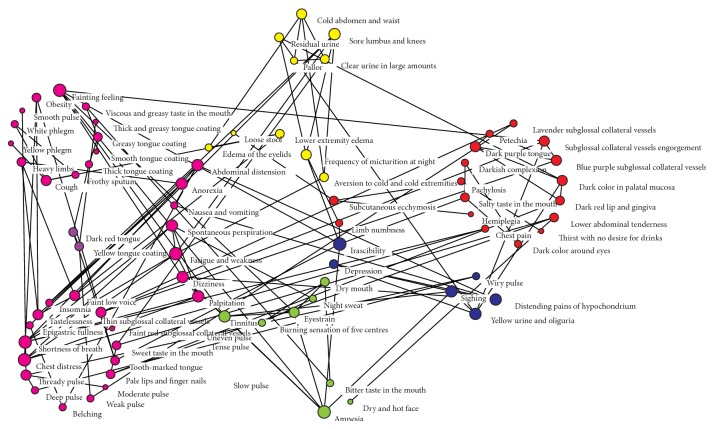
Complex characterization network node classification map of 2050 CHD patients with UAP.

The results of characterization information network properties indicate that 36 characterizations node values are 17. The maximum occupation of a characterizing information in the figure means that it has the maximum contribution to the identification of the various syndromes. Connected component represents the similarities of each node in the phenotypes network, and it means that nodes with consistent connectivity numbers are in the same network. The network with connectivity number of 1 is more complex, including 69 phenotypic nodes (Table 5, Figure 4).

### 3.5. HPO Categorization Results of 107 Pieces of Four Diagnostic Methods' Information

After comparing with items in HPO, we classified the 107 four diagnostic methods' information, following six principles. First, the appropriate characterizations were classified under the existing diseases or phenotype entries as a symptom, which took up the largest proportion of classification; for example, chest pain, chest distress, and short breath were classified under angina pectoris; cough, white phlegm, yellow phlegm, bloody phlegm, and frothy phlegm were classified under pneumonia; intolerance of cold and cold limb and burning sensation of five centers were classified under the abnormality of temperature regulation; hypodynamia and faint low voice were classified under the weakness of the classification. Second, phenotypes fully consistent with the HPO database entries could be directly classified, like palpitations, abdominal distension, depression, tinnitus, and petechiae. Third, a child node under the original entry was built, and then a lower sub-child node at next level was created under the child node, for example, establishing a child node of “abnormality of the waist” under “phenotypic abnormality”, and then creating a sub-child node of “sore waist and knee” under the “abnormality of the waist”; establishing a child node “abnormality of the perspiration” under the “abnormality of fluid regulation” and then building two sub-child nodes describing “spontaneous perspiration and night sweat” under it; Fourth, deeper descriptions for the original entries, for example, “cold sensation in abdomen” for further description about abnormality of the abdomen; “lower abdominal tenderness” made a detailed description of the location of abdominal pain; “epigastric fullness” was classified under the “abdominal distention”; bitter, sweet, salty, viscous, and greasy taste in the mouth made a detailed description of the “abnormality of taste sensation.” Fifth, items had similar meaning with original entries, for example, “irritable tantrum” and its original entry “aggressive behavior”; “sighing” and its original entry “depression”; “edema of the eyelids” and its original entry “palpebral edema”; “obesity” and its original entry “increased adipose tissue.” Sixth, description of tongue and pulse were added, in which there were 11 additional entries for pulse; 26 additional entries for tongue, and these entries were listed, respectively, in the classifications of tongue color abnormity, tongue morphological abnormalities, abnormal tongue moss, tongue ecchymosis petechiae, and abnormal sublingual collaterals.

## 4. Discussion

Tongue diagnosis is an important part of TCM inspection with a long history. It has become a very unique diagnostic method for TCM nowadays. Tongue is the seedling of heart; heart opens at tongue; the tongue is the external manifestation of heart. It is recorded in Spiritual Pivot • Meridians that “The Collateral Hand Taiyin stems and runs upward and parallel to the Channel of Hand Shaoyin into the heart. It then runs upward to link the root of tongue and pertains to the eye system.” Spiritual Pivot • Maidu records that “Heart qi goes through the tongue, only when the heart is in harmony, the tongue can taste five flavors.” Sublingual collateral diagnostic method is convenient, accurate, and noninvasive. Therefore, a lot of clinical observations of reproducibility and timeliness can be made on patients [19]. Analysis of the circulation of blood and qi by observing sublingual collaterals can provide an important basis for TCM syndrome differentiation [20]. Modern medical studies suggest that changes in blood rheology and dynamics may cause increases in sublingual vein pressure, congestion, lack of oxygen and excessive blood flow with varying degrees of varicose veins, or even collateral venous bleeding [21]. In TCM clinical researches, the length, thickness, tortuosity, and color of sublingual collaterals can serve as important auxiliary evidence to identify whether there is blood stasis pattern in patients with CHD. The sublingual collaterals of most patients with CHD share the characteristics of being long, thick, medium, or severe orthotic and with a color of purple or dark purple, which is consistent with the results of our study. In modern studies, by using inspection in combination with partial biopsy method, Ji and his team [22] found that the main manifestations of stasis are dark purple tongue, cloudy mucosa, strips or points of ecchymosis, and enlarged sublingual collaterals; microscopic test showed enlargement and congestion of capillaries in the lamina propria, red blood cell aggregation, and even the formation of microthrombus. In this aspect, they suggested that sublingual collateral abnormities are of critical significance for the diagnosis of blood stasis pattern. The first investigation on the association between traditional tongue diagnosis and the tongue coating microbiome using next-generation sequencing reveal an important connection between the tongue-coating microbiome and traditional tongue diagnosis and illustrate the potential of the tongue-coating microbiome as a novel holistic biomarker for hot or cold syndrome patient [23]. Therefore, tongue inspection is not only an indispensable objective reference for TCM clinical practice, but also a concrete external manifestation of the human body in morbid conditions.

We obtained four decision tree models of blood stasis syndrome diagnosis by extending tongue characterizations to 30 TCM four diagnostic methods' information of statistical significance and exploring with data mining methods of decision tree model. Nowadays, in modern medicine, TCM four diagnostic methods' information is seen as non-disease specific clinical manifestations and is of little significance in clinical diagnosis of Western medicine [24], while now by using decision tree method, we have extracted the most relevant data from the complex TCM four diagnostic methods' information and prompted the formation of TCM pattern diagnosis model [25, 26]. Although four kinds of blood stasis decision tree model have different characteristics, they share the same features of high specificity, sensitivity, and accuracy for the diagnosis of blood stasis. The four models selected 6-7 attributes, respectively, and the common four diagnostic methods' information were collateral color, cyanotic lips, hands, and fingernails. Therefore it can also confirm that the manifestation of sublingual collaterals is an important reference to the diagnosis of blood stasis pattern. Qu and his team found that the varicose sublingual vein is the most important attribute to identify blood stasis pattern by using data mining techniques [26]. Wang et al. found that occurrence rate of sublingual collaterals abnormities in patients with CHD was 89.29%, significantly higher than that of the control group. And with increase of age and duration of CHD, the enlargement, tortuosity, expansion, increase of collateral branches, and collateral color darkening would become worse [27]. Another study found that CHD patients had varying degrees of blood stasis manifestations, among which cyanotic lips was more common [28]. According to clinical observations, Zhang et al. believed that, in patients at primary stage of CHD accompanied by blood stasis pattern, in addition to the common chest pain, bruising and other symptoms, manifestations of cyanotic lips, complexion, hands, and fingernails could usually be seen [29].

Furthermore, we expanded 30 pieces of four diagnostic methods' information to 107 pieces. After comparing with the OMIM characterization database, there were 23 characterizations exactly consistent with each other. Results of cluster analysis showed that 21 of the 23, respectively, aggregated into five kinds of syndromes/syndrome elements and enabled the identification of qi deficiency pattern, blood stasis pattern, qi stagnation pattern, yin deficiency pattern, and yang deficiency pattern. In HPO, the use of an ontology to capture phenotypic information allows the use of computational algorithms that exploit semantic similarity between related phenotypic abnormalities to define phenotypic similarity metrics, which can be used to perform database searches for clinical diagnostics or as a basis for incorporating the human phenome into large-scale computational analysis of gene expression patterns and other cellular phenomena associated with human disease [4]. Köhler et al. consider that the differential diagnosis process is extremely challengeable due to the myriad symptoms and the extreme degree of clinical variability characteristic of many neurological diseases. Their research has demonstrated how the HPO can be incorporated into the efforts by providing a systematic semantic representation of phenotypic abnormalities encountered in human genetic diseases [30]. TCM four diagnostic methods' information also has a large quantity of features, for example, the combination of the HPO with the orphaned disease classification representing a promising resource for automated disease classification and performing computational clustering and analysis of the neurogenetic phenome. On the basis of HPO model, it is possible for TCM four diagnostic methods' characterization to identify diseases, especially TCM syndromes and symptoms, which have been preliminarily validated in the cluster analysis.

Next, we extended the sample volume of UAP cases from 385 cases to 2,050 cases, and with the application of complex network approach, we expanded 21 pieces of four diagnostic methods' characterizations to 107 pieces. There were altogether 92 characterizations being involved as components of the complex network of recognition of qi deficiency pattern, blood stasis pattern, qi stagnation pattern, and yin and yang deficiency pattern. Complex system is a network system controlled by a few hubs, with a large number of functional groups composed by hubs. It can reflect some or all of its overall characteristics or characteristics in common [31]. Robinson et al. have developed HPO with over 8000 terms representing individual phenotypic anomalies and have annotated all clinical entries in OMIM with the terms of the HPO. In his research, visualization of the human phenome was revealed and each of 727 diseases listed in OMIM for which a disorder class was defined is shown as a node in the graph and is colored according to membership in a set of 21 predefined disorder classes, defined on the basis of the physiological system [32]. Since there is a large quantity of content, numerous laws of the combinations, and various connections of TCM clinical four diagnostic methods' characterization, the traditional approach cannot reveal its connation maximally. Classification features of individual nodes in complex network, role requirements in network organization, and relationship classification of elements of network organization are potential driving force [33]. The organic combination of TCM four diagnostic methods' characterization constituted the identification elements of patterns and symptom elements, which was reflected in the connectivity of complex network. This study included a network of 69 connected nodes which indicated that the association between different four diagnostic methods' characterization was comprehensive and complex, similar to complications and accompanied syndromes in clinic. “Degree value” is the simplest but most important property of complex network nodes [34]. There were 36 characterization nodes whose degree value was 17, which contributed most to the identification of TCM patterns. Among 23 characterizations consistent with OMIM Database, the degree value of palpitation, fatigue, spontaneous perspiration, dizziness, loss of appetite, bloating, and nausea and vomiting was 17. And the other 29 nodes with highest degree value belonged to the extending characterizations, which played an important role in the identification of five kinds of syndromes diagnosis mentioned above.

The overall behavior of the system cannot be obtained only by the simple act of components (such as the functions or the features). Therefore, research methods and thinking of complex systems should be combined with when we study the syndromes and genes, proteins, and metabolomics. In addition, it should be understood that proper research ideas and research methods are usually the key points in syndrome studies. For example, a network balance model can evaluate the imbalanced network underlying TCM syndrome and find potential biomarkers [35]. A systems' biology approach with the combination of computational analysis and animal experiment is used to investigate the ZHENG (syndrome) in the context of the neuro-endocrine-immune (NEI) system, and the results demonstrate that the ZHENG (syndrome) may have a molecular basis with NEI as background [36]. HPO is able to capture phenotypic similarities between diseases in a useful and highly significant fashion. With the spread of HPO, more and more calculation methods are gradually applied in the diagnosis of human genetic diseases and finally enable the exploration of characterization data [37]. For example, groups of Köhler adapted semantic similarity metrics to measure phenotypic similarity between queries and hereditary diseases annotated with the use of the HPO and have developed a statistical model to assign *P* values to the resulting similarity scores, which can be used to rank the candidate diseases [38]. In recent years, the HPO project is collaborating with many clinical groups to refine and extend current terms and annotations. Input and collaboration from other clinical groups will be welcomed [39]. After a series of reasoning, we believe that 107 pieces of four diagnostic methods' information can be classified as the extended entry of characterization items in HPO, and based on the characteristics possessed by TCM four diagnostic methods' information, we managed to add them to HPO by six classification methods. The accurate collection, collation, and quantification of the characteristics of four diagnostic methods' information will be able to reflect the state of the body functions and characteristics of TCM disease patterns and make the TCM researches more scientific. In this study, we have only sorted the four diagnostic methods' information obtained from 107 patients with UAP. In future studies, we look forward to using HPO as research model to collect all kinds of symptoms manifested by patients under morbid conditions more comprehensively, deploying the characteristics of data mining methods. We hope to set up a large-scale, standardized database of TCM four diagnostic methods' information and realize the “high throughput analysis” based on characteristic information and then achieve the promotion of “phenomics” on systems biology.

## 5. Conclusions

After a series of reasoning, it can be believed that 107 pieces of four diagnostic methods' information can be classified as the extended entry of characterization items in HPO, and the four diagnostic methods' information of TCM is the constituent part of “phenomics.” A large-scale, standardized database of TCM four diagnostic methods' information using the HPO research model can be formed and realize the “high throughput analysis” based on characteristic information and then achieve the promotion of “phenomics” on systems biology. The concept of phenomics proposed by this paper is actually an extension of the traditionally defined “omics,” which usually refers to genomics, proteomics, transcriptomics and metabolomics, and so forth. Although the genomics research status was not involved in this study, the characteristics, such as integrity, dynamics, temporality, spatiality and complexity shared by both TCM four diagnostic methods' information and the concept of “omics” were firmly grasped by this research. HPO, based on phenomics, can be interpreted as a data-sorting and data-summarizing method for the symptoms displayed by human body in disease condition, which is in line with the connotation of the TCM four diagnostic methods' information. There is no doubt that the diversity of genome leads to the complex phenotypes, on which aspect we will do further study in the future.

## Supplementary Material

We had listed the detailed method for classification in the EXCEL after the comparison between the 107 pieces of TCM four diagnostic method's information with HPO items. There are 4 rows in the EXCEL, the first row is the Chinese Names for the “Four Diagnostic Method's Information”, the second row is the corresponding English Names for the "Four Diagnostic Method's Information", the third row is "Father Node" (the items in second row are under the third items), and the fourth row is the corresponding printscreen from HPO (http://www.human-phenotype-ontology.org/index.php/hpo_home.html), which with shading are the "Father Node". And there are 6 kinds of classified methods, which have been showed in the main content.

## Figures and Tables

**Table 1:** The distribution of sublingual collaterals of patients with CHD with different patterns (example).

Patterns	Length of collaterals			Thickness of collaterals			Tortuosity of collaterals			Colors of collaterals		
	Long	Medium	Short	Thick	Medium thick	Fine	Mild	Moderate	Severe	Mild purple	Dark	Black
Pattern of congealing cold in heart vessel	0	0	4	0	0	4	4	0	0	4	0	0
Pattern of qi deficiency and blood stasis	11	17	9	19	11	7	13	7	17	14	15	8
Pattern of mutual deficiency of qi and yin	6	9	6	7	5	9	12	2	7	15	3	3
Pattern of qi stagnation and blood stasis	4	8	0	6	4	2	5	2	5	5	4	3
Pattern of mutual obstruction of phlegm and qi	10	6	4	9	5	6	10	4	6	9	4	7
Pattern of heart vessel phlegm obstruction	1	2	7	1	3	6	10	0	0	8	2	0
Pattern of intense heart fire	0	3	0	2	0	1	3	0	0	3	0	0
Pattern of heart vessel qi stagnation	1	0	1	1	0	1	2	0	0	1	1	0
Pattern of heart qi deficiency	4	7	10	4	7	10	18	3	0	20	1	0
Heart blood stagnation pattern	26	25	12	34	12	17	32	11	20	26	26	11
Heart yang deficiency pattern	4	7	4	3	5	7	10	5	0	12	3	0
Heart yin deficiency pattern	1	0	1	0	0	2	2	0	0	2	0	0
Mutual deficiency of heart yin and yang	1	0	1	1	0	1	1	0	1	1	0	1

**Table 2:** Selected variables and corresponding values.

Selected variables	*t* test *P* value	Values	Eligible variables	*t* test *P* value	Values
Collateral color	0.000	1, 2, 3	Dark complexion	0.000	0, 1
Cyanotic lips	0.000	0, 1, 2, 3	0.5–1 cm dermal ecchymosis	0.001	0, 1
Cyanotic hands and fingernails	0.000	0, 1, 2, 3	Ecchymosis along tongue sides	0.001	0, 1
Collateral tortuosity	0.000	0, 1, 2, 3	Pale lips and fingernails	0.002	0, 1
Dark blue tongue	0.000	0, 1	Ecchymosis	0.002	0, 1
Dark red lips and gingival	0.000	0, 1	Tendency to be irritable and impatient	0.002	0, 1
Thickness of collaterals	0.000	1, 2, 3	Fatigue	0.003	0, 1, 2, 3
Length of collaterals	0.000	1, 2, 3	Laziness to speak due to qi deficiency	0.003	0, 1, 2, 3
Dark and pale complexion	0.000	0, 1	Less than 3 ecchymosis cases	0.003	0, 1
Dark Jaw mucosa	0.000	0, 1	Fissured tongue	0.003	0, 1
Shadow circled eyes	0.000	0, 1, 2, 3	Tough tongue	0.003	0, 1
Slippery pulse	0.000	0, 1	Tastelessness	0.004	0, 1, 2, 3
Loss of appetite	0.000	0, 1, 2, 3	Hesitant pulse	0.006	0, 1
Floating pulse	0.000	0, 1	Pale and dark complexion	0.007	0, 1
Soft tongue	0.000	0, 1	Subcutaneous ecchymosis	0.010	0, 1, 2, 3

*Note.* 0 = none, 1 = mild, 2 = moderate, and 3 = severe.

**Table 3:** 10-fold cross-validation of decision tree model of blood stasis pattern for 411 cases of UAP.

Algorithm	TN	FP	Sensitivity (%)	Specificity (%)	Accuracy (%)
	FN	TP			
C4.5	123	46	77.7	72.8	75.7
	54	188			

ADTree	128	41	75.6	75.7	75.7
	59	183			

CHAID	132	37	70.7	78.1	73.7
	71	171			

QUEST	60	109	93.4	35.5	69.6
	16	226			

*Note.* Sensitivity = TP/(TP + FN); Specificity = TN/(TN+ FP); Accuracy = (TP + TN)/(TP + FP + TN + FN).

**Table 4:** Characterization reference form of 23 four diagnostic methods' information.

Four diagnostic methods' information	Characterizations	Item number
Palpitation	Palpitations	7280
Aversion to cold and cold extremities	Asthenic habitus	1852
Chest pain	Chest pain	2588
Fatigue and weakness	Generalized weakness of limb muscles	4583
	Apneic episodes precipitated by illness, fatigue, stress	1796
Spontaneous perspiration	Hyperhidrosis	4890
Night sweat	Hyperhidrosis	4890
Burning sensation of five centers	Polydipsia	7753
Eyestrain	Abnormal, jerky eye movements	193
Dry mouth	Xerostomia	10302
Dizziness	Paroxysmal vertigo	7341
	Vertigo	10146
Irascibility	Irritability	5701
Scanty tongue coating	Smooth tongue	9147
Depression	Depression	3473
Anorexia	Anorexia	1445
Abdominal distension	Abdominal distention	71
Nausea and vomiting	Nausea	6834
	Nausea and vomiting	6835
Loose stool	Diarrhea	3530
Frequency of micturition at night	Nocturia	6961
Limb numbness	Cataplexy, paroxysmal weakness, or paralysis	2467
Subcutaneous ecchymosis	Ecchymoses	3790
	Petechiae	7646
Edema of the eyelids	Periorbital edema	7578
Lower extremity edema	Edema of the lower limbs	3820
Tinnitus	Tinnitus	9740

**Table 5:** Indicators of 107 characterization properties of complex networks.

Phenotypes	Values	Connected component values
Chest pain	3	1
Chest distress	17	1
Short breath	17	1
Palpitation	17	1
Cough	17	1
Aversion to cold and cold extremities	1	1
Fatigue and weakness	17	1
Spontaneous perspiration	17	1
Night sweat	2	1
Burning sensation of five centers	2	1
Eyestrain	2	1
Dry mouth	2	1
Dizziness	17	1
Amnesia	2	1
Fainting feeling	17	1
Tinnitus	2	1
Dry and hot face	2	1
Insomnia	17	1
Irascibility	4	1
Hypochondrium distending pain	4	1
Sighing	4	1
Depression	4	1
Anorexia	17	1
Abdominal distension	17	1
Epigastric fullness	17	1
Belching	17	1
Nausea and vomiting	17	1
Loose stool	1	2
Constipation	1	3
Sore lumbus and knees	1	1
Frequency of micturition at night	1	1
Limb numbness	3	1
Heel pain	1	4
Hemiplegia	3	2
Subcutaneous ecchymosis	3	2
Pachylosis	3	2
Obesity	17	1
White phlegm	17	1
Yellow phlegm	17	1
Bloody phlegm	1	5
Frothy sputum	17	1
Pharyngeal foreign body sensation	1	6
Thirst with no desire for drinks	3	2
Tastelessness	17	1
Bitter taste in the mouth	2	1
Sweet taste in the mouth	17	1
Salty taste in the mouth	3	1
Viscous and greasy taste in the mouth	17	1
Diarrhoea	1	7
Defecation asthenia	1	8
Yellow urine and oliguria	4	1
Clear urine in large amounts	1	1
Residual urine	1	1
Cold abdomen and waist	1	1
Heavy limbs	17	1
Pallor	1	1
Bright pale complexion	1	9
Darkish complexion	3	1
Sallow complexion	1	10
Red complexion	1	11
Conjunctival congestion	1	12
Dark color around eyes	3	1
Edema of the eyelids	1	1
Dark red lip and gingiva	3	1
Pale lips and finger nails	17	1
Dark color in palatal mucosa	3	1
Lower abdominal tenderness	3	1
Lower extremity edema	1	1
Faint low voice	17	1
Emaciation	0	13
Pale tongue	0	14
Red tongue	0	15
Scarlet tongue	0	16
Dark red tongue	8	1
Dark purple tongue	3	1
Swollen tongue body	0	17
Thin tongue body	0	18
Tooth-marked tongue	17	1
Thick tongue coating	17	1
Greasy tongue coating	17	1
Curdy tongue coating	0	19
Scanty tongue coating	0	20
Exfoliative tongue coating	0	21
No tongue coating	0	22
Thick and greasy tongue coating	17	1
Smooth tongue coating	17	1
Yellow tongue coating	8	1
Black tongue coating	0	23
Glossal petechia	3	1
Lavender subglossal collateral vessels	3	1
Blue purple subglossal collateral vessels	3	1
Black purple subglossal collateral vessels	0	24
Mauve subglossal collateral vessels	0	25
Faint red subglossal collateral vessels	17	1
Subglossal collateral vessels engorgement	3	1
Thin subglossal collateral vessels	17	1
Deep pulse	17	26
Rapid pulse	0	27
Moderate pulse	17	28
Knotted and intermittent pulse	0	29
Slow pulse	1	29
Thready pulse	17	26
Wiry pulse	4	26
Tense pulse	1	30
Uneven pulse	1	31
Smooth pulse	17	26
Weak pulse	17	1

## Abbreviations

TCM: Traditional Chinese medicine
HPO: Human Phenotype Ontology
OMIM: Online Mendelian Inheritance in Man
CHD: Coronary heart disease
UAP: Unstable angina pectoris
TP: True positives
TF: True false
FN: False negatives
FP: False positives.
